# WUPyV in Children with Acute Respiratory Tract Infections, China

**DOI:** 10.3201/eid1604.100011

**Published:** 2010-04

**Authors:** Xiaoyan Li, Jinying Chen, Mei Kong, Xu Su, Ming Zou, Hua Zhang, Yumin Han

**Affiliations:** Tianjin Centers for Disease Control and Prevention, Tianjin, People’s Republic of China (X. Li, M. Kong, X. Su, M. Zou); Tianjin Medical University, Tianjin (X. Li, J. Chen); Tianjin Children’s Hospital, Tianjin (H. Zhang); Tianjin Xianshuigu Hospital, Tianjin (Y. Han)

**Keywords:** Viruses, polyomavirus, WUPyV, China, respiratory tract infections, children, letter

**To the Editor:** WU polyomavirus (WUPyV) is a human polyomavirus first detected in respiratory samples in 2007 (*1*). It has since been detected worldwide (*2*–*7*), including the People’s Republic of China (*8*), but whether it is a causative respiratory pathogen remains speculative. To investigate the potential role of WUPyV in respiratory diseases and its prevalence in Tianjin, China, we examined samples obtained from children with upper respiratory tract infections (URTIs) and lower respiratory tract infections (LRTIs) by using PCR for the VP2 and LTAg genes, as described previously (*1*). As a control, we also tested samples from patients who did not have respiratory diseases.

Case-patients were 174 inpatients, hospitalized for LRTIs March–April 2008 and September 2008–February 2009, and 68 outpatients treated for URTIs November–December 2009 at Tianjin Children’s Hospital. Controls were 43 outpatients with illnesses other than respiratory diseases treated at Tianjin Xianshuigu Hospital. Most patients with LRTIs had pneumonia or bronchitis. Case-patient age ranged from 3 hours to 12 years; for 1 patient, age was unknown (Table). Nasopharyngeal aspirate samples were collected from hospitalized children, and throat swabs were obtained from outpatients with URTIs and from control children.

**Table:** Age distribution of children infected with WU polyomavirus, Tianjin, People’s Republic of China, 2008–2009*

Age	Inpatients with LRTIs	Outpatients with URTIs	Children without respiratory diseases
Mean ± SD, y	10.2 ± 16.1	5.96 ± 3.69	7.56 ± 4.25
Median age, y	0.4	5.0	7.0
Age group			
<6 mo	95 (54.6)	1 (1.5)	0
6 mo–<1 y	52 (29.9)	1 (1.5)	0
1–<2 y	15 (8.6)	3 (4.4)	2 (4.6)
2–5 y	9 (5.2)	25 (36.8)	11 (25.6)
>5 y	2 (1.1)	38 (55.9)	30 (69.8)
Unknown	1 (0.6)	0	0
Total	174 (100.0)	68 (100.0)	43 (100.0)

* LRTIs, lower respiratory tract infections; URTIs, upper respiratory tract infections. Values are no. (%) unless otherwise indicated.

Of the 174 nasopharyngeal aspirate specimens tested, 28 (16%) had WUPyV VP2 gene–positive fragments; 24 had LTAg gene–positive fragments. Four VP2-positive but LTAg-negative fragments were sequenced; nucleotide sequences were identical to WUPyV strains in GenBank. Mean age of WUPyV-infected patients was 11.7 months (range 12 days–39 months); 10 patients (36%) were <6 months of age, 10 (36%) were 6 months–1 year, 7 (25%) were 1–2 years, and 1 (4%) was 2–5 years of age. The age distribution was similar to that of the original cohort. We found WUPyV-positive samples in most months, except for March 2008. Highest proportion of WUPyV-positive samples occurred in December 2008 (27%), followed by April 2008 (25%), November 2008 (22%), and February 2009 (19%).

We detected WUPyV VP2 fragment–positive specimens by multi-PCR using the Seeplex RV Detection kit-1 (Seegene, Seoul, South Korea) for other respiratory viruses, including adenovirus, parainfluenza viruses 1, 2, 3 (PIV1, 2, 3), influenza viruses A and B, rhinovirus (rhinovirus V), human metapneumovirus, respiratory syncytial virus A and B (RSV A, B), and coronaviruses OC43/HKU1 and 229E/NL63, according to the manufacturers’ instructions. First strands of cDNA were produced by using the RevertAid First Strand cDNA Synthesis Kit (Fermentas, Glen Burnie, MD, USA). Human bocavirus (HBoV) was tested as described previously (*9*).

Twenty (71%) case-patients were co-infected with other respiratory viruses, most commonly RSV B (9/28, 32%), followed by HBoV (6/28, 21%), rhinovirus V and PIV3 (4 each of 28, 14%), human metapneumovirus (3/28, 11%), adenovirus and influenza A (2 each of 28, 7%), and 229E (1/28, 4%). Of 20 patients with co-infections, 14 (50%) were infected with 2 viruses; 2 (7%) with 3 viruses (WU, RSV B, PIV3; and WU, influenza virus, A, HBoV); 3 (11%), with 4 viruses (WU, RSV B, PIV3, influenza A; WU, RSV B, PIV3, HBoV; and WU, RSV B, rhinovirus V, HBoV); and 1 (4%), with 5 viruses (WU, RSV B, PIV3, BoV, rhinovirus V).

Three (4%) of 68 throat swabs from outpatients with URTIs were WUPyV positive, substantially lower than from inpatients with LRTIs. Among 3 WUPyV-positive case-patients, 2 were 2 years of age and 1 was 3. No WUPyV was detected in 43 control samples.

The prevalence of WUPyV in hospitalized children with acute respiratory tract infections in Tianjin was 16.1%, higher than 7.1% found in a study in the United States (*3*); 4.9% in Germany (*4*); 4.5% in Australia (*5*); 6.29% in Thailand (*6*); and 2.2% in Lanzhou, China (*8*). Variation may be due to different geographic and age distributions of the virus. Another study reported that frequencies of WUPyV in URTIs (6.7%) and LRTIs cases (7.1%) were comparable (*10*). However, we found the incidence of WUPyV in patients with LRTIs (16.1%) was higher than in patients with URTIs (4.4%). Among WUPyV-infected patients with LRTIs, 71.4% were <1 year of age, which was comparable to populations investigated in other studies (*2*,*3*,*6*). Although ≈60% of outpatients with URTIs were >5 years of age, none was WUPyV positive. This finding suggests WUPyV may play a major role in young children, especially infants, with LRTIs.

Most WUPyV infection has been detected during later winter and early spring (*2*,*4*,*5*) although other research showed no seasonal distribution (*6*). We found 2 peaks, in April and December 2008 (L. Xiaoyan et al., unpub. data). We also detected 1 WUPyV-infected case in September 2008, which suggests WUPyV could also occur in summer months.

Frequency of WUPyV co-infection with other pathogens varied from 42.1% to 79.7% (*4*–*6*). Although we showed a co-infection rate of 71.4%, there were 8 (28.6%) of 28 patients with respiratory illness in whose specimens we detected only 1 virus, WUPyV. No WUPyV was detected in samples from 43 control patients, whereas in patients with LRTIs and URTIs, infection rates were 16.1% and 4.4%, respectively. These findings suggest WUPyV may be a potential pathogenic agent in children with acute respiratory tract infections. More comprehensive case–control investigations are needed to determine the association of WUPyV infections with respiratory diseases.
